# Translation and cultural adaptation of “Glasgow Children’s Benefit Inventory” into Brazilian Portuguese

**DOI:** 10.1016/j.bjorl.2023.101353

**Published:** 2023-10-23

**Authors:** Caroline Catherine Lacerda Elias, Adriane Ribeiro Teixeira, Maria Eduarda Claro de Souza, Letícia Petersen Schmidt Rosito, Sady Selaimen da Costa

**Affiliations:** a Hospital Nossa Senhora da Conceição (HNSC), Porto Alegre, RS, Brazil; b Universidade Federal do Rio Grande do Sul, Porto Alegre, RS, Brazil

**Keywords:** Quality of life, Postoperative period, Otolaryngology, Translation, Cross-cultural studies

## Abstract

**Objective:**

Translate and cross-culturally adapt into Brazilian Portuguese the Glasgow Children’s Benefit Inventory instrument used for the quality-of-life assessment after pediatric ENT interventions.

**Method:**

This is a methodological study of translation and cross-cultural adaptation of the GCBI instrument following seven stages: 1) Translation of two versions by two independent translators, 2) Elaboration of a consensual synthetized version, 3) Assessment of the synthetized version by experts, 4) Assessment by the target audience, 5) Back-translation, 6) Pilot study and 7) Use of the instrument. The final version of the instrument was answered by a sample of 28 people responsible for children aged from 2 to 7 years, submitted to tonsillectomy between January 2019 and December 2021, in a public hospital in Porto Alegre. The collection considered patients with a minimum of 6-months and a maximum of 3-years of postoperative follow-up.

**Result:**

The instrument final version was compared to the original version showing semantic equivalence, absence of consistent translation difficulties and appropriate cross-cultural adaptation, and well understood by the target audience. The application of the questionnaire in the sample showed a Cronbach alpha coefficient of 0.944 corresponding to a high degree of reliability of the instrument.

**Conclusion:**

The translation and cross-cultural adaptation showed semantic appropriateness and its use when assessing ENT postoperative results in a pediatric population showed high reliability of the instrument.

**Level of evidence:**

4.

## Highlights

• The GCBI questionnaire in tonsillectomized children obtained a Cronbach's alpha of 0.94.
• The translation of the GCBI questionnaire maintained semantic equivalence.
• The cross-cultural adaptation of the GCBI is conceptually and culturally adequate for the sample.

## Introduction

The quality-of-life assessment tools are fundamental for the analysis of different treatments and medical conditions since they measure global changes in the health condition of patients and allow comparisons with other clinical conditions.^1^ It has been demonstrated that measurement instruments of postoperative results in health, used in a systematic way, result in a better communication and decision-making between doctors and patients and increase satisfaction of patients with health care.^2^ The verification of health-related quality of life of pediatric patients helps healthcare professionals to understand the impact caused by illness or treatment on the patient’s life.^3^ Thus, it is important to have validated instruments in Brazilian Portuguese available for the health-related quality life assessment of children.

The literature offers a series of questionnaires used to measure pediatric ENT postoperative results, but most of the instruments assess the global quality of life of the patient without correlating it with an improvement or worsening after a specific intervention. The most frequently used instruments are the “Pediatric Quality of Life Questionnaire”, the “KINDL-R Questionnaire” to measure the health-related quality of life in children and teenagers, the “Child Health Questionnaire”, the “Child Behavior Checklist”, the “Preschool children quality of life questionnaire” and the “Glasgow Children’s Benefit Inventory (GCBI)”, as the review carried out.^1^ Among the analyzed questionnaires, the only one projected to be sensitive to changes after a medical intervention or treatment was the “Glasgow Children Benefit Inventory”, widely used to assess the impact of different procedures (cochlear implant,^4^ bone anchored prosthesis,^5^ nasal endoscopic surgery,^6^ adenotonsillectomy^7^). It was originally described in English and has already been validated in several languages, such as German,^7^, ^8^, ^9^, ^10^ Italian,^11^ Dutch,^12^ Spanish,^13^ Swedish,^14^ Greek,^6^ Turkish,^15^ Russian^16^ and Mandarin Chinese,^17^ allowing the comparison of results obtained from ENT interventions and treatments between different countries.

This study was carried out due to the importance of measurement tools, translated and adapted into Brazilian Portuguese, for the assessment of the impact on the quality of life of children under 12 years old after pediatric ENT interventions. We chose the GCBI because it was specifically developed for this purpose and is widely used in the international literature, what allows comparisons of results.^18^, ^19^ Thus, the objectives of the present study were to translate and cross-culturally adapt the GCBI questionnaire.

## Method

This methodological study was conducted, after the authors of the original instrument have authorized it’s translation into Brazilian Portuguese, in accordance with the ethical norms and was approved by the Research Ethics Committee under the number CAAE 42075520300005327. All participants digitally signed the Free and Informed Consent Term.

The sample chosen for the application of the questionnaire was of children submitted to tonsillectomy, a common surgical procedure in pediatric population for either sleep apnea syndrome or chronic infection, alterations that significantly impact the quality of life of patients^20^

The study was developed in 7-stages as proposed by Borsa et al.^21^:

1) Translation into Portuguese: Translation into Portuguese by two independent bilingual translators (semantic equivalence).
2) Synthesis of the translated versions: Synthesis of the two translations by the researchers.
3) Evaluation of the synthesis by experts: Translated and synthetized version evaluated by a committee formed by five experts. After the independent analysis and together with the committee, an adapted version was obtained with the necessary changes for a better interpretation.
4) Evaluation by the target audience: Evaluation of the translated questionnaire by 10 individuals from the target audience. Convenience sampling was selected considering patients submitted to tonsillectomy at a university hospital. A survey of patients under the following inclusion criteria was required to the hospital department in charge: children up to the age of 12 under tonsillectomy postoperative follow-up (including patients who were submitted to minor nasal procedures at the same surgical time such as adenoidectomy and/or cauterization of the inferior turbinates) between January 2019 and December 2021 with a minimum of 6 months (considering the period of time enough for surgical recovery) up to a maximum of 3 years of postoperative care (to mitigate memory bias). Exclusion criteria: patients submitted to tympanostomy with ventilation tube placement at the same surgical time to avoid confounding bias relating to postoperative satisfaction. Data from the patients’ records was used for further information about the diagnosis and the intervention to which they had been submitted.

The caregivers of selected children were contacted and participated via phone call (up to 3 phone call attempts were made at different times and on different days).

5) Back translation: The synthetized and reviewed version used with the target audience was back translated into the original language (English) by two new translators. Afterwards, a synthesis of the two back translated versions was carried out by the researchers, reflecting the content of items according to the original version, presenting a conceptual equivalence.
6) Pilot study: Pilot collection of the final version of the questionnaire was carried out with 10 participants, in a self-applied way, without the researcher’s interference, via digital message app. All participants answered all the questions and did not make any contact mentioning difficulties and, therefore, no changes were made in this stage.
7) Application of the adapted instrument: For the calculation of the sample size to estimate the Cronbach alpha coefficient the PSS Health (Power and Sample Size for Health Researchers) tool was used. Considering an instrument with 24 items, margin of error of 0.1, confidence level of 95% and expected Cronbach alpha coefficient of 0.92 as referred by Kubba et al.,^19^ a sample size of 24 subjects was determined.

Sample collection of the instrument for the analysis of internal consistency was carried out through the Cronbach alpha coefficient.

### Glasgow Children’s Benefit Inventory (Appendix 1)

The questionnaire comprises 24 questions and was retrospectively answered by those responsible for the children after the intervention. In addition to a general result, it contains four assessment subscales: psychosocial (Questions 8, 11, 17, 18, 19), physical health (Questions 1, 14, 22, 23, 24), behavior (Questions 3, 4, 9, 12, 13, 15, 16) and vitality (Questions 2, 5, 6, 7, 10, 21).

The response options in the questionnaire are listed using Likert-type scales with 5 options. After the application, responses must be rescaled as follows^18^: −100 (maximum damage), −50 (moderate damage), 0 (no change), +50 (moderate benefit) and +100 (maximum benefit). The total scale and subscales are obtained by calculating the average of responses to the items.

### Statistical analysis

Data was exported from Google Forms software, where the questionnaire was answered, to Microsoft Excel software.

The statistical analyses were performed by the software SPSS version 21.0. The results obtained were described in the sample by the median and the interquartile range while the internal consistency of items was evaluated by the Cronbach alpha coefficient.

## Results

Each question was analyzed as shown in Table 1, comparing the original questionnaire (Column 1) with the synthetized translation (Column 2). After the analysis by the group of experts and target audience, the final version was generated (Column 3) and, based on it, the back translated version was made (Column 4). The changes made in the translation and cultural adaptation of the questionnaire can be found in Column 5.

**Table 1:** Linguistic adaptations of the GCBI to the Portuguese language spoken in Brazil.

Original Questionnaire	Synthesized Translation	Final Version Questionnaire	Synthesized Back-Translation	Cross-cultural Adaptation
Glasgow children’s benefit inventory	Avaliação de Glasgow dos benefícios a criança	Avaliação de Glasgow dos benefícios à criança	Glasgow questionnaire for benefit assessment on kids	“Inventory” foi traduzido para “avaliação” para melhor compreensão.
In this questionnaire, we are interested to know how much change you think there has been in your child’s general condition since his or her operation.	Com este questionário, queremos saber quantas mudanças você acha que houve na condição geral de seu filho (a) desde a operação.	Com este questionário, queremos saber o quanto a condição geral de seu filho (a) mudou desde a cirurgia dele (a).	This questionnaire aims to assess how much of your child's general condition has changed since the operation.	“how much change you think there has been” foi traduzido para “o que mudou” para simplificar a sentença.
1. Has your child’s operation made his/her overall life better or worse?	1. A operação de seu/sua filho (a) melhorou ou piorou a vida dele (a) em geral?	1. A cirurgia de seu/sua filho (a) melhorou ou piorou a vida dele (a) em geral?	1. Has your child’s operation improved or worsen their overall life?	Realizado juste na concordância verbal das respostas.
Much better	Muito melhor	Melhorou muito	Improved a lot	
A little better	Um pouco melhor	Melhorou um pouco	Improved somewhat	
No change	Não mudou	Não mudou	No change	
A little worse	Um pouco pior	Piorou um pouco	Worsened somewhat	
Much worse	Muito pior	Piorou muito	Worsened a lot	
2. Has your child’s operation affected the things he/she does?	2. A operação de seu/sua filho (a) afetou as atividades diárias dele(a)?	2.A cirurgia de seu/sua filho (a) afetou as atividades diárias dele (a)?	2. Has your child's operation affected their daily activities?	Realizado ajuste na concordância verbal das respostas.
Much better	Muito melhor	Melhoraram muito	A lot better	“Things he/she does” foi traduzida para “atividades diárias dele/dela” para melhor interpretação.
A little better	Um pouco melhor	Melhoraram um pouco	Somewhat better	
No change	Não mudou	Não mudou	No change	
A little worse	Um pouco pior	Pioraram um pouco	Somewhat worse	
Much worse	Muito pior	Pioraram muito	A lot worse	
3. Has your child’s operation made his/her behavior better or worse?	3. A operação de seu/sua filho (a) melhorou ou piorou o comportamento dele (a)?	3. A cirurgia de seu/sua filho (a) melhorou ou piorou o comportamento dele (a)?	3. Has your child's operation improved or worsened their behavior?	Realizado ajuste na concordância verbal das respostas
Much better	Muito melhor	Melhorou muito	Improved a lot	
A little better	Um pouco melhor	Melhorou um pouco	Improved somewhat	
No change	Não mudou	Não mudou	No change	
A little worse	Um pouco pior	Piorou um pouco	Worsened somewhat	
Much worse	Muito pior	Piorou muito	Worsened a lot	
4. Has your child’s operation affected his/her progress and development?	4. A operação de seu/sua filho (a) afetou o progresso e desenvolvimento dele (a)?	4. A cirurgia de seu/sua filho (a) afetou o progresso e desenvolvimento dele (a)?	4. Has your child's operation affected their progress/development?	Realizado ajuste na concordância verbal das respostas
Much better	Muito melhor	Melhorou muito	Improved a lot	
A little better	Um pouco melhor	Melhorou um pouco	Improved somewhat	
No change	Não mudou	Não mudou	No change	
A little worse	Um pouco pior	Piorou um pouco	Worsened somewhat	
Much worse	Muito pior	Piorou muito	Worsened a lot	
5.Has your child’s operation affected how lively he/she is during the day?	5. A operação de seu/sua filho (a) afetou a disposição dele(a) durante o dia?	5. A cirurgia de seu/sua filho (a) afetou a disposição dele (a) durante o dia?	5. Has your child's operation affected their liveliness during the day?	Expressão utilizada na pergunta foi incorporada no corpo da resposta para deixar as opções de resposta mais completas e claras.
Much better	Muito melhor	Está muito mais disposto (a)	The child has a lot more liveliness	
A little better	Um pouco melhor	Está um pouco mais disposto (a)	The child has more liveliness	
No change	Não mudou	Não mudou	No change	
A little worse	Um pouco pior	Está um pouco menos disposto (a)	The child has less liveliness	
Much worse	Muito pior	Está bem menos disposto (a)	The child has a lot less liveliness	
6. Has your child’s operation affected how well he/she sleeps at night?	6 – A operação de seu/sua filho (a) afetou a qualidade do sono dele (a)?	6. A cirurgia de seu/sua filho (a) afetou a qualidade do sono dele (a)?	6. Has your child's operation affected their sleep quality?	Realizado ajuste na concordância verbal das respostas
Much better	Muito melhor	Melhorou muito	Improved a lot	“How well he/she sleeps at night” foi traduzida como “qualidade do sono dele(a)”, para melhor compreensão.
A little better	Um pouco melhor	Melhorou um pouco	Improved somewhat	
No change	Não mudou	Não mudou	No change	
A little worse	Um pouco pior	Piorou um pouco	Worsened somewhat	
Much worse	Muito pior	Piorou muito	Worsened a lot	
7. Has your child’s operation affected his/her enjoyment of food?	7. A operação de seu/sua filho (a) afetou o gosto dele (a) pelos alimentos?	7. A cirurgia de seu/sua filho (a) afetou o prazer dele (a) ao se alimentar?	7. Has your child's operation affected their eating pleasure?	“his/her enjoyment of food” foi substituído por “o prazer dele(a) ao se alimentar”, com mais fácil entendimento
Much better	Muito melhor	Aumentou muito	Improved a lot	
A little better	Um pouco melhor	Aumentou um pouco	Improved somewhat	
No change	Não mudou	Não mudou	No change	
A little worse	Um pouco pior	Diminuiu um pouco	Decreased somewhat	
Much worse	Muito pior	Diminuiu muito	Decreased a lot	
8. Has your child’s operation affected how self-conscious he/she is with other people?	8. A operação de seu/sua filho (a) afetou o quão autoconsciente ele (a) é em relação a outras pessoas?	8. A cirurgia de seu/sua filho (a) afetou o quão tímido ele se sente em relação a outras pessoas?	8. Has your child's operation affected how shy they feel towards other people?	Expressão utilizada na pergunta foi incorporada no corpo da resposta para deixar as opções de resposta mais completas e claras.
Much better	Muito melhor	Muito menos tímido	A lot less shy	“how self-conscious he/she is with other people” foi adaptada para “o quão tímido ele(a) se sente em relação a outras pessoas”, expressão mais utilizada no Brasil.
A little better	Um pouco melhor	Um pouco menos tímido	Somewhat less shy	
No change	Não mudou	Não mudou	No change	
A little worse	Um pouco pior	Um pouco mais tímido	Somewhat more shy	
Much worse	Muito pior	Muito mais tímido	A lot more shy	
9. Has your child’s operation affected how well he/she gets on with the rest of the family?	9.A operação de seu/sua filho (a) afetou a maneira como ele (a) se relaciona com o resto da família?	9. A cirurgia de seu/sua filho (a) mudou a maneira como ele (a) se relaciona com o resto da família?	9. Has your child's operation changed how they deal with other family members?	Realizado ajuste na concordância verbal nas respostas
Much better	Muito melhor	Melhorou muito	Improved a lot	“how well he/she gets on with the rest of the family” foi traduzido para “a maneira como ele(a) se relaciona com o resto da família”, elucidando melhor o sentido da expressão.
A little better	Um pouco melhor	Melhorou um pouco	Improved somewhat	
No change	Não mudou	Não mudou	No change	
A little worse	Um pouco pior	Piorou um pouco	Worsened somewhat	
Much worse	Muito pior	Piorou muito	Worsened a lot	
10. Has your child’s operation affected his/her ability to spend time and have fun with friends?	10. A operação de seu/sua filho (a) afetou a habilidade dele (a) de passar tempo e divertir-se com amigos?	10. A cirurgia de seu/sua filho (a) afetou a habilidade dele (a) de passar tempo e divertir-se com amigos?	10. Has your child's operation affected their ability to spend time and have fun with friends?	Realizado ajuste na concordância verbal das respostas
Much better	Muito melhor	Melhorou muito	Improved a lot	
A little better	Um pouco melhor	Melhorou um pouco	Improved somewhat	
No change	Não mudou	Não mudou	No change	
A little worse	Um pouco pior	Piorou um pouco	Worsened somewhat	
Much worse	Muito pior	Piorou muito	Worsened a lot	
11. Has your child’s operation affected how embarrassed he/she is with other people?	11. A operação de seu/sua filho(a) afetou o constrangimento dele(a) perante outras pessoas?	11. A cirurgia de seu filho (a) afetou o quanto ele (a) se sente constrangido (a) quando está com outras pessoas?	11. Has your child's operation affected how self-conscious they feel?	Expressão utilizada na pergunta foi incorporada no corpo da resposta para deixar as opções de resposta mais completas e claras.
Much better	Muito melhor	Muito menos constrangido	A lot less self-conscious	“embarrassed” foi substituída por “constrangido(a)”, adequando ao vocabulário local
A little better	Um pouco melhor	Um pouco menos constrangido	Somewhat less self-conscious	
No change	Não mudou	Não mudou	No change	
A little worse	Um pouco pior	Mais constrangido	More self-conscious	
Much worse	Muito pior	Muito mais constrangido	A lot more self-conscious	
12. Has your child’s operation affected how easily distracted he/she has been?	12. A operação de seu/sua filho (a) afetou a facilidade com que ele(a) se distrai?	12. A cirurgia de seu/sua filho (a) afetou a facilidade com que ele (a) se distrai?	12. Has your child's operation affected how easily distracted they get?	Expressão utilizada na pergunta foi incorporada no corpo da resposta para deixar as opções de resposta mais completas e claras.
Much better	Muito melhor	Se distrai muito menos	A lot less distracted	
A little better	Um pouco melhor	Se distrai um pouco menos	Somewhat less distracted	
No change	Não mudou	Não mudou	No change	
A little worse	Um pouco pior	Se distrai um pouco mais	Somewhat more distracted	
Much worse	Muito pior	Se distrai muito mais	A lot more distracted	
13.Has your child’s operation affected his/her learning?	13. A operação de seu/sua filho(a) afetou a aprendizagem dele(a)?	13. A cirurgia de seu/sua filho (a) afetou a aprendizagem dele (a)?	13. Has your child's operation affected their learning?	Realizado ajuste na concordância verbal nas respostas
Much better	Muito melhor	Melhorou muito	Improved a lot	
A little better	Um pouco melhor	Melhorou um pouco	Improved somewhat	
No change	Não mudou	Não mudou	No change	
A little worse	Um pouco pior	Piorou um pouco	Worsened somewhat	
Much worse	Muito pior	Piorou muito	Worsened a lot	
14. Has your child’s operation affected the amount of time he/she has had to be off nursery, playgroup or school?	14. A operação de seu/sua filho(a) afetou o tempo que ele(a) ficou fora da creche, escolinha ou escola?	14. A cirurgia de seu/sua filho (a) afetou a quantidade de tempo que ele (a) teve que faltar à creche, escolinha ou escola?	14. Has your child's operation affected how often they had to skip school?	Expressão utilizada na pergunta foi incorporada no corpo da resposta para deixar as opções de resposta mais completas e claras.
Much better	Muito melhor	Falta muito menos	A lot less often	“had to be off” foi traduzida como “teve que faltar” adequando ao vocabulário
A little better	Um pouco melhor	Falta menos	Less often	
No change	Não mudou	Não mudou	No change	
A little worse	Um pouco pior	Falta mais	More often	
Much worse	Muito pior	Falta muito mais	A lot more often	
15. Has your child’s operation affected his/her ability to concentrate on a task?	15. A operação de seu/sua filho (a) afetou a habilidade dele(a) em se concentrar em uma tarefa?	15. A cirurgia de seu/sua filho (a) afetou a habilidade dele (a) em se concentrar em uma tarefa?	15. Has your child's operation affected their ability to focus on a task?	Expressão utilizada na pergunta foi incorporada no corpo da resposta para deixar as opções de resposta mais completas e claras.
Much better	Muito melhor	Se concentra muito mais	A lot more focused	
A little better	Um pouco melhor	Se concentra mais	More focused	
No change	Não mudou	Não mudou	No change	
A little worse	Um pouco pior	Se concentra menos	Less focused	
Much worse	Muito pior	Se concentra muito menos	A lot less focused	
16. Has your child’s operation affected how frustrated and irritable he/she is?	16. A operação de seu/sua filho (a) afetou a frustração e irritação dele (a)?	16. A cirurgia de seu/sua filho (a) afetou o quão frustrado (a) e irritado ele (a) fica?	16. Has your child's operation affected how frustrated/irritable they get?	Expressão utilizada na pergunta foi incorporada no corpo da resposta para deixar as opções de resposta mais completas e claras.
Much better	Muito melhor	Está muito menos frustrado (a) e irritado (a)	A lot less frustrated/irritable	“is” foi substituída por “fica” para ajustar ao contexto
A little better	Um pouco melhor	Está menos frustrado (a) e irritado (a)	Less frustrated/irritable	
No change	Não mudou	Não mudou	No change	
A little worse	Um pouco pior	Está mais frustrado (a) e irritado (a)	More frustrated/irritable	
Much worse	Muito pior	Está muito mais frustrado (a) e irritado (a)	A lot more frustrated/irritable	
17. Has your child’s operation affected how he/she feels about himself/herself?	17. A operação de seu/sua filho (a) afetou os sentimentos dele (a) sobre si mesmo?	17. A cirurgia de seu/sua filho (a) afetou os sentimentos dele (a) sobre si mesmo?	17. Has your child's operation affected their self-esteem?	Expressão utilizada na pergunta foi incorporada no corpo da resposta para deixar as opções de resposta mais completas e claras.
Much better	Muito melhor	Está se sentindo muito melhor	Self-esteem is a lot better	
A little better	Um pouco melhor	Está se sentindo um pouco melhor	Self-esteem is somewhat better	
No change	Não mudou	Não mudou	No change	
A little worse	Um pouco pior	Está se sentindo um pouco pior	Self-esteem is somewhat worse	
Much worse	Muito pior	Está se sentindo muito pior	Self-esteem is a lot worse	
18. Has your child’s operation affected how happy and content he/she is?	18. A operação de seu/sua filho(a) afetou o quão feliz e contente ele(a) se sente?	18. A cirurgia de seu/sua filho (a) afetou o quão feliz e contente ele (a) se sente?	18. Has your child's operation affected how happy they feel?	Expressão utilizada na pergunta foi incorporada no corpo da resposta para deixar as opções de resposta mais completas e claras.
Much better	Muito melhor	Está muito mais feliz e contente	A lot happier	
A little better	Um pouco melhor	Está um pouco mais feliz e contente	Somewhat happier	
No change	Não mudou	Não mudou	No change	
A little worse	Um pouco pior	Está um pouco menos feliz e contente	Somewhat less happy	
Much worse	Muito pior	Está muito menos feliz e contente	A lot less happy	
19. Has your child’s operation affected his/her confidence?	19. A operação de seu/sua filho(a) afetou a autoconfiança dele(a)?	19. A cirurgia de seu/sua filho (a) afetou a autoconfiança dele (a)?	19. Has your child's operation affected their self-confidence?	Expressão utilizada na pergunta foi incorporada no corpo da resposta para deixar as respostas mais completas e claras.
Much better	Muito melhor	Está muito mais autoconfiante	A lot more self-confident	“confidence” foi traduzido como “autoconfiança” para melhor interpretação
A little better	Um pouco melhor	Está um pouco mais autoconfiante	Somewhat more self-confident	
No change	Não mudou	Não mudou	No change	
A little worse	Um pouco pior	Está um pouco menos autoconfiante	Somewhat less self-confident	
Much worse	Muito pior	Está muito menos autoconfiante	A lot less self-confident	
20. Has your child’s operation affected his/her ability to care for himself/herself as well as you think they should, such as washing, dressing and using the toilet?	20. A operação de seu/sua filho (a) afetou a habilidade dele (a) em cuidar de si mesmo (a) da forma que você acha que ele (a) deveria como lavar-se, vestir-se e usar o banheiro?	20. A cirurgia de seu/sua filho (a) afetou a habilidade dele (a) em cuidar de si mesmo (a) da forma que você acha que ele (a) deveria (lavar-se, vestir-se e usar o banheiro)?	20. Has your child's operation affected their ability to self-care, such as taking a shower or using the bathroom?	
Much better	Muito melhor	Está muito melhor	A lot better	
A little better	Um pouco melhor	Está um pouco melhor	Somewhat better	
No change	Não mudou	Não mudou	No change	
A little worse	Um pouco pior	Está um pouco pior	Somewhat worse	
Much worse	Muito pior	Está muito pior	A lot worse	
21.Has your child’s operation affected his/her ability to enjoy leisure activities such as swimming and sports, and general play?	21. A operação de seu/sua filho (a) afetou a habilidade de ele (a) curtir atividades de lazer como natação e esportes, bem como brincadeiras em geral?	21. A cirurgia de seu/sua filho (a) afetou a habilidade de ele (a) aproveitar atividades de lazer como natação e esportes, bem como brincadeiras em geral?	21. Has your child's operation affected their ability to enjoy activities such as swimming, sports, and games in general?	Expressão utilizada na pergunta foi incorporada no corpo da resposta para deixar as opções de resposta mais completas e claras.
Much better	Muito melhor	Está aproveitando muito mais	Enjoying a lot more	
A little better	Um pouco melhor	Está aproveitando um pouco mais	Enjoying somewhat more	
No change	Não mudou	Não mudou	No change	
A little worse	Um pouco pior	Está aproveitando um pouco menos	Enjoying somewhat less	
Much worse	Muito pior	Está aproveitando muito menos	Enjoying a lot less	
22. Has your child’s operation affected how prone he/she is to catch colds or infections?	22. A operação de seu/sua filho(a) afetou a propensão dele(a) em ficar resfriado ou pegar alguma infecção?	22. A cirurgia de seu/sua filho (a) mudou a frequência em que ele (a) fica resfriado (a) ou tem alguma infecção?	22. Has your child's operation changed how often they catch a cold or infection?	Expressão utilizada na pergunta foi incorporada no corpo da resposta para deixar as opções de resposta mais completas e claras.
Much better	Muito melhor	Ficou muito menos propenso à infecção	A lot less prone to catching infections	“how prone” foi substituído por a “frequência” facilitando entendimento da frase
A little better	Um pouco melhor	Ficou menos propenso à infecção	Somewhat less prone to catching infections	
No change	Não mudou	Não mudou	No change	
A little worse	Um pouco pior	Ficou mais propenso à infecção	More prone to catching infections	
Much worse	Muito pior	Ficou muito mais propenso à infecção	A lot more prone to catching infections	
23. Has your child’s operation affected how often he/she needs to visit a doctor?	23. A operação de seu/sua filho(a) afetou a frequência em que ele necessita ir ao médico?	23. A cirurgia de seu/sua filho (a) afetou a frequência com que ele necessita ir ao médico?	23. Has your child's operation affected how often they need to visit a doctor?	expressão utilizada na pergunta foi incorporada no corpo da resposta para deixar as opções de resposta mais completas e claras.
Much better	Muito melhor	Necessita ir muito menos	A lot less often	
A little better	Um pouco melhor	Necessita ir um pouco menos	Somewhat less often	
No change	Não mudou	Não mudou	No change	
A little worse	Um pouco pior	Necessita ir um pouco mais	Somewhat more often	
Much worse	Muito pior	Necessita ir muito mais	A lot more often	
24. Has your child’s operation affected how much medication he/she has needed to take?	24. A operação de seu/sua filho(a) afetou a quantidade de remédios que seu/sua filho(a) tem precisado tomar?	24. A cirurgia de seu/sua filho (a) afetou a quantidade de remédios que seu/sua filho (a) tem precisado tomar?	24. Has your child's operation affected the amount of medicine they need?	expressão utilizada na pergunta foi incorporada no corpo da resposta para deixar as opções de resposta mais completas e claras.
Much better	Muito melhor	Tem precisado de muito menos remédios	Needs a lot less medicine	
A little better	Um pouco melhor	Tem precisado de um pouco menos remédios	Needs less medicine	
No change	Não mudou	Não mudou	No change	
A little worse	Um pouco pior	Tem precisado de um pouco mais remédios	Needs somewhat more medicine	
Much worse	Muito pior	Tem precisado de muito mais remédios	Needs a lot more medicine	

The final version of the translated and adapted instrument in Brazilian Portuguese can be found in Appendix 2.

For the collection of responses obtained in the questionnaire, a phone call attempt was made to 99 people responsible for the operated children (up to 3 attempts were made on different days and at different times). Among the 99 people contacted, 69 (69.6%) accepted the phone invitation to participate in the study, 29 (29.2%) did not answer the phone call and 1 (1.1%) refused to participate. The Free and Informed Consent Term and the adapted questionnaire were sent via a digital message app for the self-application of those 69 participants who agreed to be part of the study.

**Figure 1:**
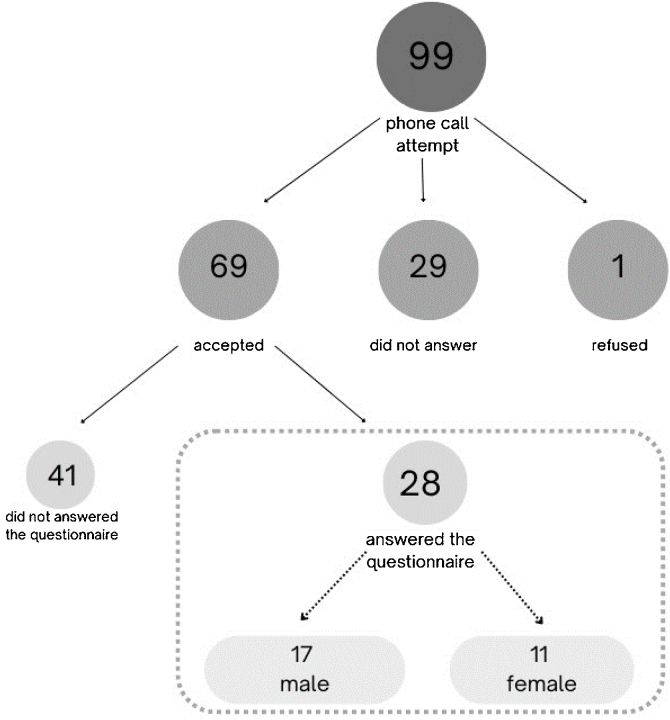
Sample selection flowchart.

A total of 28 (40.5%) responded questionnaires were returned, which were used for the data analysis. In this sample, there was information from responsible people for 17 male children and 11 female children submitted to tonsillectomy. The ages of patients varied from 2-years old to 7-years old on the date of the surgery with an average of 4-years old (Fig. 1).

Considering the 24 questions, a total Cronbach alpha coefficient of 0.94 was found, what suggests a high reliability of the items.

In the sample, it was also found in the result of the questionnaire general score a median of +66.7 (responses with interquartile range between +48.43 and +95.31), what suggests an improvement in the postoperative quality of life.

**Figure 2:**
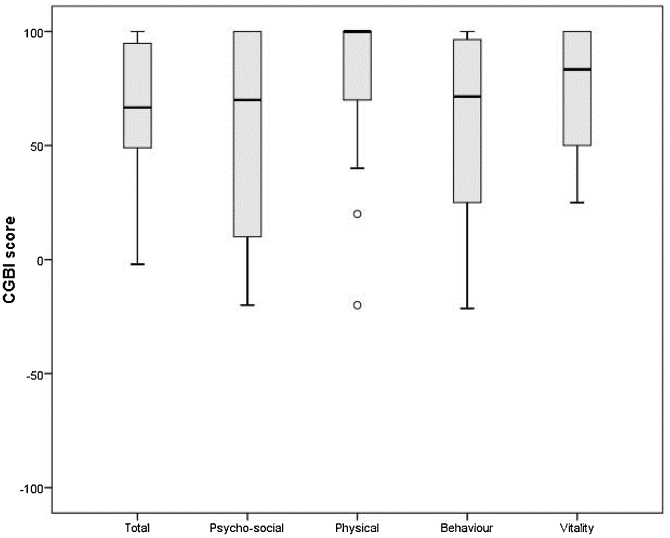
Glasgow Children’s Benefits Inventory Total score and subscales (psychosocial, physical health, behavior, and vitality) for patients submitted to tonsillectomy (n = 24). The box plots show the interquartile ranges, the thicker line shows the median. Values higher than 1.5 times the interquartile range are shown as circles.

In the result of the subareas evaluated by the questionnaire, positive results were also observed, what suggests a postoperative improvement. In the psychosocial evaluation, a median of +70 was found (responses with interquartile range between +5 and +100), in the physical health evaluation a median of +100 was obtained (responses with an interquartile range between +70 and +100), in the analysis of behavior a median of +71.42 was found (responses with an interquartile range between +23.21 and +98.21), in the subarea of vitality a median of +83.33 was observed (responses with an interquartile range between +50 and +100) (Fig. 2).

## Discussion

In this article we presented the translation and cultural adaptation into Brazilian Portuguese of the questionnaire “Glasgow Children’s Benefit Inventory”. We applied the questionnaire with those responsible for children submitted to tonsillectomy under 12 years old in a retrospective way so that the measurement of changes in the quality of life could be more sensitive.

Although there are various methods of translation and cultural adaptation, we chose the one proposed by Borsa et al.^21^ The method encompasses the internationally known concept of back translation and details the process of cultural adaptation and has been used by many authors for both translation and cultural adaptation.^22^, ^23^

It is important to make it clear that the translation process is the first stage of the cultural adaptation process. After translating from the original language to the target language, we observe the characteristics of the instrument that have semantic equivalence and at the same time adapt, linguistic and culturally, to a different contexto.^24^ Two independent translators made two versions of the questionnaire (Stage 1) that could be compared and discussed in the elaboration of a synthetized version (Stage 2) by the researchers. That mitigates the risks of linguistic, psychological, cultural as well as theoretical and practical understanding biases.^25^

The cultural adaptation that resulted from the evaluation of the translated version when compared with the original version by five experts, both independently and together (Stage 3) demonstrated appropriate results of understanding of the text during the application with the target audience (Sage 4). The terminology of the instrument was understandable in all items of the questionnaire and did not arise any question from the target audience.

After the back translation (Stage 5) of the final version a small difference in the expressions used was observed when compared to the original version. Changes in expressions and grammar structures of some items were made based on the need to obtain semantic, idiomatic, experimental, and conceptual equivalence, which was nearly total between the two versions. Such changes can be observed in Column 5 of Table 1.

The final version of the instrument turned out to be conceptually and culturally appropriate for the target audience.

We observed the questionnaire is retrospectively applied and there may be a memory bias. The author of the original instrument justifies this choice in the form of application of the questionnaire with the purpose of obtaining a higher sensitivity in the detection of changes when directing questions about the variation observed in the health condition due to the intervention, instead of taking pre and postoperative actions and subtracting one from the Other.^19^

Even though the authors^19^ described in the original instrument that it is appropriate for a broad pediatric age group, we suggest it should be applied with caution in children under 2-years old due to the possible difficulty of interpretation of some items for infants. In this study, questionnaires were not applied in children under two years old.

During the application for the assessment of children submitted to tonsillectomy, the participation rate of respondents to the questionnaire was of 40.5%, similar to the participation rate of 38%^19^ in the original study. The analysis of responses showed high reliability of the items, evidenced by the Cronbach alpha coefficient of 0.94, similar to the original article, which obtained a Cronbach alpha of 0.92^19^ and similar to the translations and cultural adaptations into Arabic^4^ 0.9 and German^8^ 0.84.

We suggest the continuity of the instrument analysis, an increase in the sample for application of psychometric tests and validation of the questionnaire.

## Conclusion

The translation and cross-cultural adaptation conducted made it possible the creation of a Brazilian version of the Glasgow Children’s Benefit Inventory for the assessment of children’s quality of life after ENT interventions. The final version of the adapted instrument, named Avaliação de Glasgow dos Benefícios à Criança is semantically, conceptually, and culturally equivalent to the original and applicable to the Brazilian children. Its application in children submitted to tonsillectomy showed high reliability of the items.

## Funding

No funding.

## Conflicts of interest

The authors declare no conflicts of interest.
