# Supplementary material for: Translation and cultural adaptation of “Glasgow Children’s Benefit Inventory” into Brazilian Portuguese
Source: Braz J Otorhinolaryngol. 2023 Oct 23;90(1):101353. doi: 10.1016/j.bjorl.2023.101353 (PMC10711033; doi:10.1016/j.bjorl.2023.101353)
Supplement: Supplementary file 1 [file mmc1.docx]

**BJORL-D-23-00080_Supplementary material**

**Appendix 1** **Glasgow Children’s Benefit Inventory.** In this questionnaire, we are interested to know how much change you think there has been in your child’s general condition since his or her operation.

| 1. Has your child’s operation made his/her overall life better or worse? | | | | |
| --- | --- | --- | --- | --- |
| Much better | A little better | No change | A little worse | Much worse |
| 2. Has your child’s operation affected the things he/she does? | | | | |
| Much better | A little better | No change | A little worse | Much worse |
| 3. Has your child’s operation made his/her behaviour better or worse? | | | | |
| Much better | A little better | No change | A little worse | Much worse |
| 4. Has your child’s operation affected his/her progress and development? | | | | |
| Much better | A little better | No change | A little worse | Much worse |
| 5. Has your child’s operation affected how lively he/she is during the day? | | | | |
| Much better | A little better | No change | A little worse | Much worse |
| 6. Has your child’s operation affected how well he/she sleeps at night? | | | | |
| Much better | A little better | No change | A little worse | Much worse |
| 7. Has your child’s operation affected his/her enjoyment of food? | | | | |
| Much better | A little better | No change | A little worse | Much worse |
| 8. Has your child’s operation affected how self-conscious he/she is with other people? | | | | |
| Much better | A little better | No change | A little worse | Much worse |
| 9. Has your child’s operation affected how well he/she gets on with the rest of the family? | | | | |
| Much better | A little better | No change | A little worse | Much worse |
| 10. Has your child’s operation affected his/her ability to spend time and have fun with friends? | | | | |
| Much better | A little better | No change | A little worse | Much worse |
| 11. Has your child’s operation affected how embarrassed he/she is with other people? | | | | |
| Much better | A little better | No change | A little worse | Much worse |
| 12. Has your child’s operation affected how easily distracted he/she has been? | | | | |
| Much better | A little better | No change | A little worse | Much worse |
| 13. Has your child’s operation affected his/her learning? | | | | |
| Much better | A little better | No change | A little worse | Much worse |
| 14. Has your child’s operation affected the amount of time he/she has had to be off nursery, playgroup, or school? | | | | |
| Much better | A little better | No change | A little worse | Much worse |
| 15. Has your child’s operation affected his/her ability to concentrate on a task? | | | | |
| Much better | A little better | No change | A little worse | Much worse |
| 16. Has your child’s operation affected how frustrated and irritable he/she is? | | | | |
| Much better | A little better | No change | A little worse | Much worse |
| 17. Has your child’s operation affected how he/she feels about himself/herself? | | | | |
| Much better | A little better | No change | A little worse | Much worse |
| 18. Has your child’s operation affected how happy and content he/she is? | | | | |
| Much better | A little better | No change | A little worse | Much worse |
| 19. Has your child’s operation affected his/her confidence? | | | | |
| Much better | A little better | No change | A little worse | Much worse |
| 20. Has your child’s operation affected his/her ability to care for himself/herself as well as you think they should, such as washing, dressing and using the toilet? | | | | |
| Much better | A little better | No change | A little worse | Much worse |
| 21. Has your child’s operation affected his/her ability to enjoy leisure activities such as swimming and sports, and general play? | | | | |
| Much better | A little better | No change | A little worse | Much worse |
| 22. Has your child’s operation affected how prone he/she is to catch colds or infections? | | | | |
| Much better | A little better | No change | A little worse | Much worse |
| 23. Has your child’s operation affected how often he/she needs to visit a doctor? | | | | |
| Much better | A little better | No change | A little worse | Much worse |
| 24. Has your child’s operation affected how much medication he/she has needed to take? | | | | |
| Much better | A little better | No change | A little worse | Much worse |

**Appendix 2 Avaliação de Glasgow dos Benefícios à Criança.** Com este questionário, queremos saber o quanto a condição geral de seu filho (a) mudou desde a cirurgia dele (a).

| 1. A cirurgia de seu/sua filho (a) melhorou ou piorou a vida dele (a) em geral? |
| --- |
| Melhorou muito |
| Melhorou um pouco |
| Não mudou |
| Piorou um pouco |
| Piorou muito |
| 2. A cirurgia de seu/sua filho (a) afetou as atividades diárias dele (a)? |
| Melhoraram muito |
| Melhoraram um pouco |
| Não mudou |
| Pioraram um pouco |
| Pioraram muito |
| 3. A cirurgia de seu/sua filho (a) melhorou ou piorou o comportamento dele (a)? |
| Melhorou muito |
| Melhorou um pouco |
| Não mudou |
| Piorou um pouco |
| Piorou muito |
| 4. A cirurgia de seu/sua filho (a) afetou o progresso e desenvolvimento dele (a)? |
| Melhorou muito |
| Melhorou um pouco |
| Não mudou |
| Piorou um pouco |
| Piorou muito |
| 5. A cirurgia de seu/sua filho (a) afetou a disposição dele (a) durante o dia? |
| Está muito mais disposto (a) |
| Está um pouco mais disposto (a) |
| Não mudou |
| Está um pouco menos disposto (a) |
| Está bem menos disposto (a) |
| 6. A cirurgia de seu/sua filho (a) afetou a qualidade do sono dele (a)? |
| Melhorou muito |
| Melhorou um pouco |
| Não mudou |
| Piorou um pouco |
| Piorou muito |
| 7. A cirurgia de seu/sua filho (a) afetou o prazer dele (a) ao se alimentar? |
| Aumentou muito |
| Aumentou um pouco |
| Não mudou |
| Diminuiu um pouco |
| Diminuiu muito |
| 8. A cirurgia de seu/sua filho (a) afetou o quão tímido ele se sente em relação a outras pessoas? |
| Muito menos tímido |
| Um pouco menos tímido |
| Não mudou |
| Um pouco mais tímido |
| Muito mais tímido |
| 9. A cirurgia de seu/sua filho (a) mudou a maneira como ele (a) se relaciona com o resto da família? |
| Melhorou muito |
| Melhorou um pouco |
| Não mudou |
| Piorou um pouco |
| Piorou muito |
| 10. A cirurgia de seu/sua filho (a) afetou a habilidade dele (a) de passar tempo e divertir-se com amigos? |
| Melhorou muito |
| Melhorou um pouco |
| Não mudou |
| Piorou um pouco |
| Piorou muito |
| 11. A cirurgia de seu filho (a) afetou o quanto ele (a) se sente constrangido (a) quando está com outras pessoas? |
| Muito menos constrangido |
| Um pouco menos constrangido |
| Não mudou |
| Mais constrangido |
| Muito mais constrangido |
| 12. A cirurgia de seu/sua filho (a) afetou a facilidade com que ele (a) se distrai? |
| Se distrai muito menos |
| Se distrai um pouco menos |
| Não mudou |
| Se distrai um pouco mais |
| Se distrai muito mais |
| 13. A cirurgia de seu/sua filho (a) afetou a aprendizagem dele (a)? |
| Melhorou muito |
| Melhorou um pouco |
| Não mudou |
| Piorou um pouco |
| Piorou muito |
| 14. A cirurgia de seu/sua filho (a) afetou a quantidade de tempo que ele (a) teve que faltar à creche, escolinha ou escola? |
| Falta muito menos |
| Falta menos |
| Não mudou |
| Falta mais |
| Falta muito mais |
| 15. A cirurgia de seu/sua filho (a) afetou a habilidade dele (a) em se concentrar em uma tarefa? |
| Se concentra muito mais |
| Se concentra mais |
| Não mudou |
| Se concentra menos |
| Se concentra muito menos |
| 16. A cirurgia de seu/sua filho (a) afetou o quão frustrado (a) e irritado ele (a) fica? |
| Está muito menos frustrado (a) e irritado (a) |
| Está menos frustrado (a) e irritado (a) |
| Não mudou |
| Está mais frustrado (a) e irritado (a) |
| Está muito mais frustrado (a) e irritado (a) |
| 17. A cirurgia de seu/sua filho (a) afetou os sentimentos dele (a) sobre si mesmo? |
| Está se sentindo muito melhor |
| Está se sentindo um pouco melhor |
| Não mudou |
| Está se sentindo um pouco pior |
| Está se sentindo muito pior |
| 18. A cirurgia de seu/sua filho (a) afetou o quão feliz e contente ele (a) se sente? |
| Está muito mais feliz e contente |
| Está um pouco mais feliz e contente |
| Não mudou |
| Está um pouco menos feliz e contente |
| Está muito menos feliz e contente |
| 19. A cirurgia de seu/sua filho (a) afetou a autoconfiança dele (a)? |
| Está muito mais autoconfiante |
| Está um pouco mais autoconfiante |
| Não mudou |
| Está um pouco menos autoconfiante |
| Está muito menos autoconfiante |
| 20. A cirurgia de seu/sua filho (a) afetou a habilidade dele (a) em cuidar de si mesmo (a) da forma que você acha que ele (a) deveria (lavar-se, vestir-se e usar o banheiro)? |
| Está muito melhor |
| Está um pouco melhor |
| Não mudou |
| Está um pouco pior |
| Está muito pior |
| 21. A cirurgia de seu/sua filho (a) afetou a habilidade de ele (a) aproveitar atividades de lazer como natação e esportes, bem como brincadeiras em geral? |
| Está aproveitando muito mais |
| Está aproveitando um pouco mais |
| Não mudou |
| Está aproveitando um pouco menos |
| Está aproveitando muito menos |
| 22. A cirurgia de seu/sua filho (a) mudou a frequência em que ele (a) fica resfriado (a) ou tem alguma infecção? |
| Ficou muito menos propenso à infecção |
| Ficou menos propenso à infecção |
| Não mudou |
| Ficou mais propenso à infecção |
| Ficou muito mais propenso à infecção |
| 23. A cirurgia de seu/sua filho (a) afetou a frequência com que ele necessita ir ao médico? |
| Necessita ir muito menos |
| Necessita ir um pouco menos |
| Não mudou |
| Necessita ir um pouco mais |
| Necessita ir muito mais |
| 24. A cirurgia de seu/sua filho (a) afetou a quantidade de remédios que seu/sua filho (a) tem precisado tomar? |
| Tem precisado de muito menos remédios |
| Tem precisado de um pouco menos remédios |
| Não mudou |
| Tem precisado de um pouco mais remédios |
| Tem precisado de muito mais remédios |
